# Nicotine up-regulates SLC7A5 expression depending on TRIM29 in non-small cell lung cancer

**DOI:** 10.1016/j.gendis.2023.04.016

**Published:** 2023-06-16

**Authors:** Dahua Liu, Haolin Ren, Guimin Wen, Pu Xia

**Affiliations:** a Biological Anthropology Institute, College of Basic Medical Science, Jinzhou Medical University, Jinzhou, Liaoning 121000, China; b Department of Radiology, The First Affiliated Hospital of Dalian Medical University, Dalian, Liaoning 116011, China; c Department of Community Nursing, College of Nursing, Jinzhou Medical University, Jinzhou, Liaoning 121000, China

Non-small cell lung cancer (NSCLC) is a malignant tumor that poses a serious threat to human health. Adenocarcinoma (LUAD) and squamous cell carcinoma (LUSC) are two common types of NSCLC. They originate from glandular and squamous cells of the bronchial epithelium, respectively. Identifying gene changes during tumorigenesis is conducive to the selection of suitable treatment methods for patients. CD98 is a heterodimeric transmembrane glycoprotein, which is composed of a heavy chain and a light chain.^1^ CD98 heavy chain (CD98hc), also known as 4F2hc or SLC3A2, is an 85 kDa type II transmembrane glycoprotein, which consists of a cytoplasmic region (NH2 terminal), a single chain transmembrane region, and a huge extracellular region (COOH terminal).^1^ CD98hc can combine with the CD98 light chain, LAT1 (SLC7A5), to form CD98 protein through disulfide bonds.^1^ SLC7A5 is a 12 times transmembrane helix bundle protein composed of 501–535 amino acid residues.^1^ The overexpression of CD98 is closely related to the occurrence and development of NSCLC. Therefore, we screened the expression profiles of SLC3A2 and SLC7A5 in smoking and non-smoking patients with LUSC and LUAD. In addition, we attempted to determine the mechanisms underlying nicotine-induced SLC7A5 expression in LUSC and LUAD cells.

The mRNA levels of SLC3A2 and SLC7A5 in patients with LUAD and LUSC with different smoking habits were analyzed using The Cancer Genome Atlas (TCGA) database. Compared with matched normal tissues, SLC3A2 and SLC7A5 mRNA levels were higher in LUAD and LUSC tissues (Fig. S1A, B). No difference in SLC3A2 expression was observed between smoking and non-smoking LUSC or LUAD patients (Fig. S1C). SLC7A5 expression was higher in smoking patients with LUAD than in non-smoking patients (Fig. S1D). After screening the differentially expressed genes in the NSCLC cohort in the TCGA database, 660 genes with differential expression were identified between SLC3A2 high and low groups, including 446 up-regulated genes and 214 down-regulated genes, while 409 up-regulated genes and 210 down-regulated genes were identified between SLC7A5 high and low groups. Heatmaps and volcano curves were used to visualize the different genes in each group (Fig. S2, 3A, B). Kyoto Encyclopedia of Genes and Genomes (KEGG) pathway analysis was performed based on up-regulated and down-regulated genes. Up-regulated genes were mainly enriched in metabolism, whereas down-regulated genes were related to immune function (Fig. S2, 3C). Gene Ontology (GO) enrichment analysis demonstrated that the up-regulated genes were mainly enriched for chromosome segregation, nuclear division, and chromosome separation, whereas the down-regulated genes were mainly enriched in immune responses, tissue homeostasis, and INF-γ responses (Fig. S2, 3D). These data confirmed that SLC3A2 and SLC7A5 function together as a whole. Correlation analysis showed that epidermal growth factor receptor (EGFR) was positively correlated with TRIM29 and NFKB1, and negatively correlated with SLC3A2, SLC7A5, and SERPINB5 in smoking patients with LUAD, while it was positively correlated with SLC3A2 and SLC7A5 in non-smoking patients (Fig. 1A). The correlation between EGFR and TRIM29 was reversed in smoking and non-smoking patients with LUAD (Fig. 1A). This indicates that nicotine may be a factor that changes SLC7A5 expression through TRIM29 in LUAD patients. In both smoking and non-smoking LUSC patients, these genes were positively correlated with each other (Fig. 1A). EGFR, SERPINB5, TRIM29, and NFKB1 were expressed at higher levels in LUSC patients than in LUAD patients (Fig. S4A). Particularly, lower TRIM29 expression was observed in LUAD tumoral tissues than in matched normal tissues (Fig. S4A). LUAD patients with low TRIM29 expression had a better survival rate than those with high TRIM29 expression, while LUSC patients showed the opposite trend (Fig. S4B). Next, we analyzed the potential mechanisms of nicotine-regulated SLC7A5 expression in NSCLC based on TRIM29. The results of the colony formation assay showed that nicotine induced proliferation of both A549 and H226 cells (Fig. S5A). TRIM29 knockdown offset the effects of nicotine on the two cell lines (Fig. S5A). Knockdown of MYB or SLC7A5 inhibited the proliferation of A549 and H226 cells (Fig. S5B, C). Western blotting results showed that nicotine induced EGFR, SERPINB5, TRIM29, and SLC7A5 expression and NFKB1 phosphorylation in A549 and H226 cells (Fig. 1B). TRIM29 knockdown suppressed nicotine-induced SLC7A5 expression (Fig. 1C). MYB knockdown inhibited SLC7A5 expression in A549 and H226 cells (Fig. 1D). The potential binding site of the SLC7A5 sequence to MYB was predicted using JASPAR (Fig. S6). The DNA (SLC7A5)–protein (MYB) complex was observed using EMSA (Fig. 1E). These results indicated an interaction between SLC7A5 and MYB proteins. This indicated a potential molecular mechanism of MYB-regulated SLC7A5 expression.

**Figure 1:**
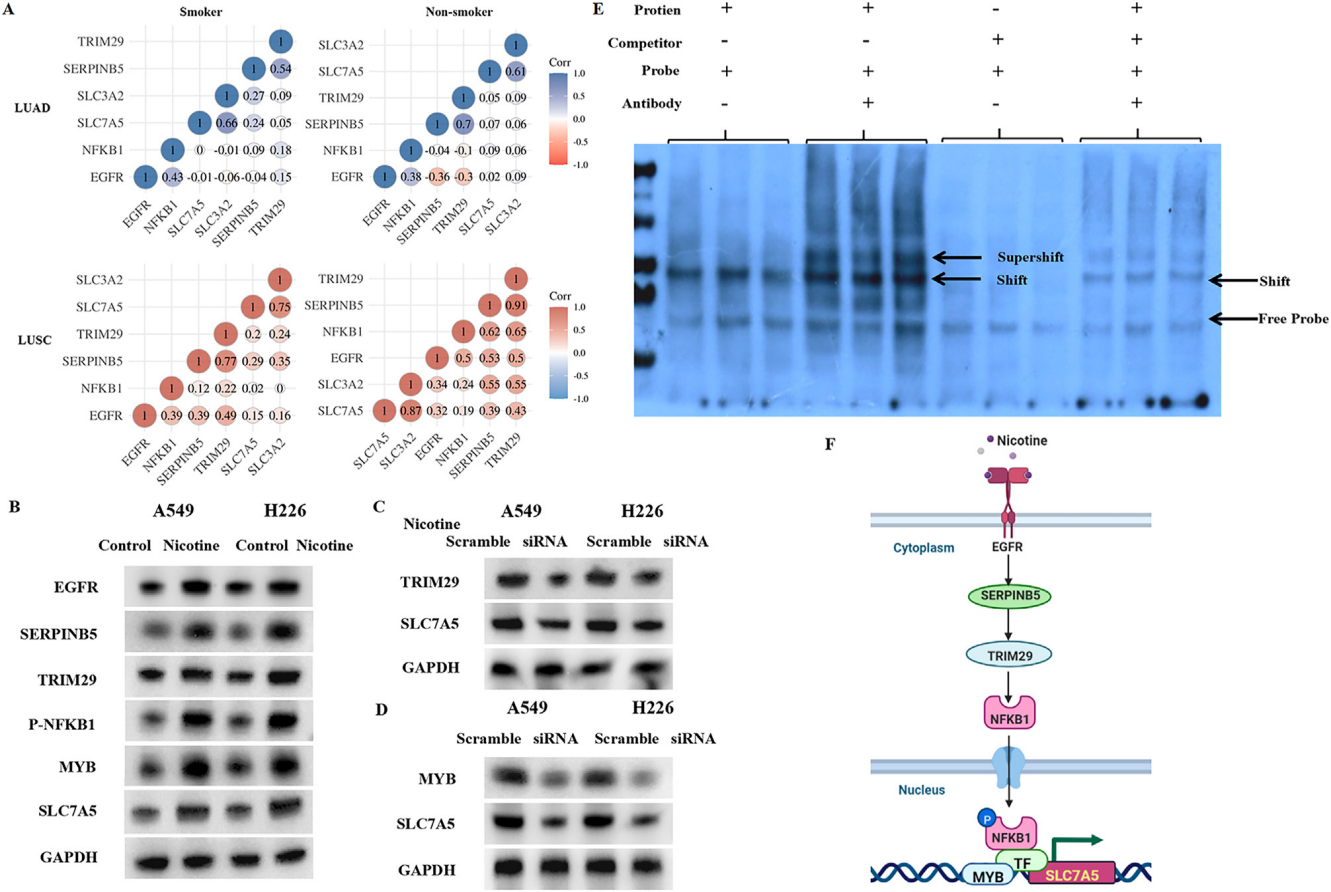
The mechanisms of nicotine-induced SLC7A5 expression in NSCLC patients and cells. **(A)** Correlation genes of SLC7A5 in smoking and non-smoking patients with LUAD and LUSC. **(B)** The levels of SLC7A5 correlated proteins in A549 cells and H226 cells after nicotine treatment. **(C)** The SLC7A5 protein level in TRIM29 knockdown A549 cells and H226 cells after nicotine treatment. **(D)** The SLC7A5 protein level in MYB knockdown A549 cells and H226 cells. **(E)** DNA (SLC7A5)-protein (MYB) complex (“shift”) and DNA (SLC7A5)-protein (MYB)-antibody complex (“supershift”) were detected using EMSA. **(F)** Schematic diagram of the potential mechanisms of nicotine-induced SLC7A5 expression in NSCLC.

In this study, we first found the differential expression of SLC7A5 in LUAD patients with different smoking statuses. Smoking is closely related to lung cancer, especially squamous cell carcinoma. Cigarette smoke contains more than 3000 toxic chemicals and 70 carcinogens, including nicotine. Repeated stimulation of bronchial mucosa or glands by carcinogens increases the risk of lung cancer. To our knowledge, no previous study has demonstrated the influence of nicotine on SLC7A5 expression in lung cancer cells. We confirmed that nicotine induced the proliferation and SLC7A5 expression in LUSC and LUAD cells. Previous studies have demonstrated that nicotine stimulates epidermal growth factor (EGF) secretion and results in EGFR expression to induce the tumorigenesis of lung cancer.^2^ In our bioinformatic analysis, we confirmed that TRIM29 was positively correlated with EGFR in smoking patients with LUAD and negatively correlated with EGFR in non-smoking patients. Interestingly, nicotine did not induce the proliferation of TRIM29 knockdown LUSC and LUAD cells. TRIM29, also known as the ataxia group D complementary gene (ATDC), is highly expressed in NSCLC tissues and an unfavorable prognostic marker for NSCLC patients.^3^ This evidence shows that TRIM29 is a link between EGFR and SLC7A5 in lung cancer cells. Therefore, we examined the upstream and downstream genes of TRIM29 that can be linked with EGFR and SLC7A5. We found that nicotine induced EGFR expression and the next expression of SERPINB5 also increased. SERPINB5 has been identified as a hub gene in lung cancer patients.^4^ Lung cancer patients with a high SERPINB5 expression had a poorer overall survival time than those with a low SERPINB5 expression.^4^ In the downstream of TRIM29, we confirmed that NF-κB was activated after TRIM29 overexpression in lung cancer cells. These results are consistent with those of previous studies on lung cancer and bladder cancer. The transcriptional regulation of c-Myb, which is mediated by the NF-κB/P–TEFb complex, has been confirmed in osteosarcoma.^5^ We also found the up-regulation of c-Myb after NF-κB pathway activation. In addition, we confirmed the interaction between the SLC7A5 DNA sequence and c-Myb. This provides a deeper understanding of c-Myb's regulation on SLC7A5 expression in lung cancer cells.

In this study, we focused on the differential expression of SLC7A5 in smoking and non-smoking patients with LUAD. Mechanistically, we found a complete pathway to explain nicotine-induced SLC7A5 expression in NSCLC cells for the first time. Nicotine-induced EGFR expression in NSCLC cells and SERPINB5 and TRIM29 were subsequently overexpressed, which activated the NF-κB pathway and the transcriptional regulation of c-Myb. This pathway can explain the mechanism of nicotine-induced SCL7A5 expression in NSCLC cells (Fig. 1F). Our data provide new therapeutic targets for nicotine-associated lung cancer.

## Author contributions

PX conceived and planned the project. PX conducted the experiments, supervised the study, and wrote the manuscript. DHL and HLR completed the bioinformatics analysis and figure generation. DHL and GMW conducted the experiments and analyzed and discussed the results. All authors read and approved the final manuscript.

## Conflict of interests

The authors declare no conflict of interests.

## Funding

This study was supported by the 10.13039/100014717National Natural Scientific Foundation of China (No. 81972784) and the “Double First-Class” Disciplinary Construction Project of Jinzhou Medical University (Liaoning, China).
