# Supplementary material for: Nicotine up-regulates SLC7A5 expression depending on TRIM29 in non-small cell lung cancer
Source: Genes Dis. 2023 Jun 16;11(2):582–4. doi: 10.1016/j.gendis.2023.04.016 (PMC10491962; doi:10.1016/j.gendis.2023.04.016)
Supplement: Multimedia component 1 [file mmc1.docx]

**Supplementary Data**

**Supplementary material and methods**

**Materials and methods**

**Bioinformatic analysis**

LUAD and LUSC mRNA expression data (HTSeq FPKM) and corresponding clinical data were downloaded from TCGA database (http://cancergenome.nih.gov/). Perl programming was used to integrate the data into gene expression matrix. R programming Limma package was used to check the different expression genes between tumor tissues and normal tissues. The survival status, age, sex, TNM stage, T stage, N stage, M stage and other clinical information of patients were extracted for subsequent analysis. R programming Survival package was used for univariate and multivariate Cox analysis to evaluate the relationship between age, gender, TNM stage, T stage, N stage, M stage and prognosis of the patients. Gene Ontology (GO) enrichment was performed to investigate the functions of SLC3A2 or SLC7A5 and the Kyoto Encyclopedia of Genes and Genomes (KEGG) pathway enrichment was conducted to explore the potential molecular mechanisms of the differentially expressed SLC3A2 or SLC7A5. The correlation map of EGFR, SERPINB5, TRIM29, NFKB1 and SLC7A5 was constructed by R programming ggstatsplot package.

**Cell culture**

Lung adenocarcinoma cells, A549, and lung squamous cell carcinoma cells, H226, were purchased from the American Type Culture Collection (ATCC, Bethesda, MD) and grown in DMEM medium (Hyclone, Logan, UT) supplemented with 10% fetal bovine serum and antibiotics (100 U/ml penicillin and 100 ug/ml streptomycin).

**TRIM29 knockdown and nicotine treatment**

The oligoribonucleotide sequence of the negative control, human TRIM29 siRNA 1, and human TRIM29 siRNA 2 as follows: 5’-UUCUCCGAACGUGUCACGUTT-3’, 5’-GAGCUGCGCAAGUCCAUUUTT-3’, and 5’-ACGGAGCUGUCAUUGCAAATT-3’ were synthesized by Sangon Biotech Co., Ltd (Shanghai, China). A mixture of siRNA 1, siRNA 2 and RNAiMax (Invitrogen, Shanghai, China) was added to medium at a final siRNA concentration of 100  nmol/l following the manufacturer’s protocol. Cells were used for experiments after siRNA transfection 48 h. Cells with or without TRIM29 knockdown were treated with 0.5 µM nicotine to keep a constant concentration for 48h before experiments.

**MYB or SLC7A5 knockdown**

The transfection methods and reagents were same as TRIM29 knockdown. siRNAs for silencing of SLC7A5 (sc-62555), c-Myb (sc-29855) and a negative control (sc-37007) were purchased from Santa Cruz Biotechnology (Shanghai, China).

**Colony formation assay**

Cells (1×10^3^) were plated in 24-well plates. After two weeks, colonies were fixed with 4% formaldehyde and stained with 1% crystal violet. The number of colonies was counted manually.

**Western blot**

Cellular protein (30 μg) was separated with 8% sodium dodecylsulfate polyacrylamide gel electrophoresis (SDS-PAGE) gel and transferred to polyvinylidene fluoride (PVDF) membranes. The membranes were then incubated in 5% milk for 2h at room temperature and with the first antibody overnight at 4°C. Primary antibodies were EGFR (sc-373746, Santa Cruz), SERPINB5 (sc-166260, Santa Cruz), TRIM29 (sc-166718, Santa Cruz), P-NFKB1 (sc-271908, Santa Cruz), c-Myb (sc-74512, Santa Cruz), SLC7A5 (sc-47724, Santa Cruz), and GAPDH (sc-74512, Santa Cruz). After 24h, the membranes were incubated with secondary antibodies for 2h at room temperature. The signals were detected using an enhanced chemiluminescence kit (Beyotime Biotechnology, Shanghai, China).

**Transcription factor binding site prediction**

The possible binding sites of MYB on SLC7A5 oligonucleotide was predicted by JASPAR database. JASPAR (https://jaspar.genereg.net) is an open-access database of curated, non-redundant transcription factor (TF) binding profiles stored as position frequency matrices (PFMs) and TF flexible models (TFFMs) for TFs across multiple species in six taxonomic groups [1].

**Electrophoretic Mobility Shift Assays (EMSA)**

Gel shifts were performed with an oligonucleotide of SLC7A5 containing a consensus heterodimeric binding site for MYB. Cell lysate in the presence or absence of MYB was incubated with the ^32^P-labeled oligonucleotide probe and subjected to EMSA. The oligonucleotide-protein complexes were separated and transferred to nylon membrane. Competition assays were performed using unlabeled double stranded DNA that was added to the reaction before the addition of oligonucleotide probe. Supershift was observed using MYB antibody.

**Supplementary references**

1. Castro-Mondragon JA, Riudavets-Puig R, Rauluseviciute I, Lemma RB, Turchi L, Blanc-Mathieu R, Lucas J, Boddie P, Khan A, Manosalva Pérez N, Fornes O, Leung TY, Aguirre A, Hammal F, Schmelter D, Baranasic D, Ballester B, Sandelin A, Lenhard B, Vandepoele K, Wasserman WW, Parcy F, Mathelier A. JASPAR 2022: the 9th release of the open-access database of transcription factor binding profiles. Nucleic Acids Res. 2022;50(D1):D165-D173.

**Supplementary figures**

**
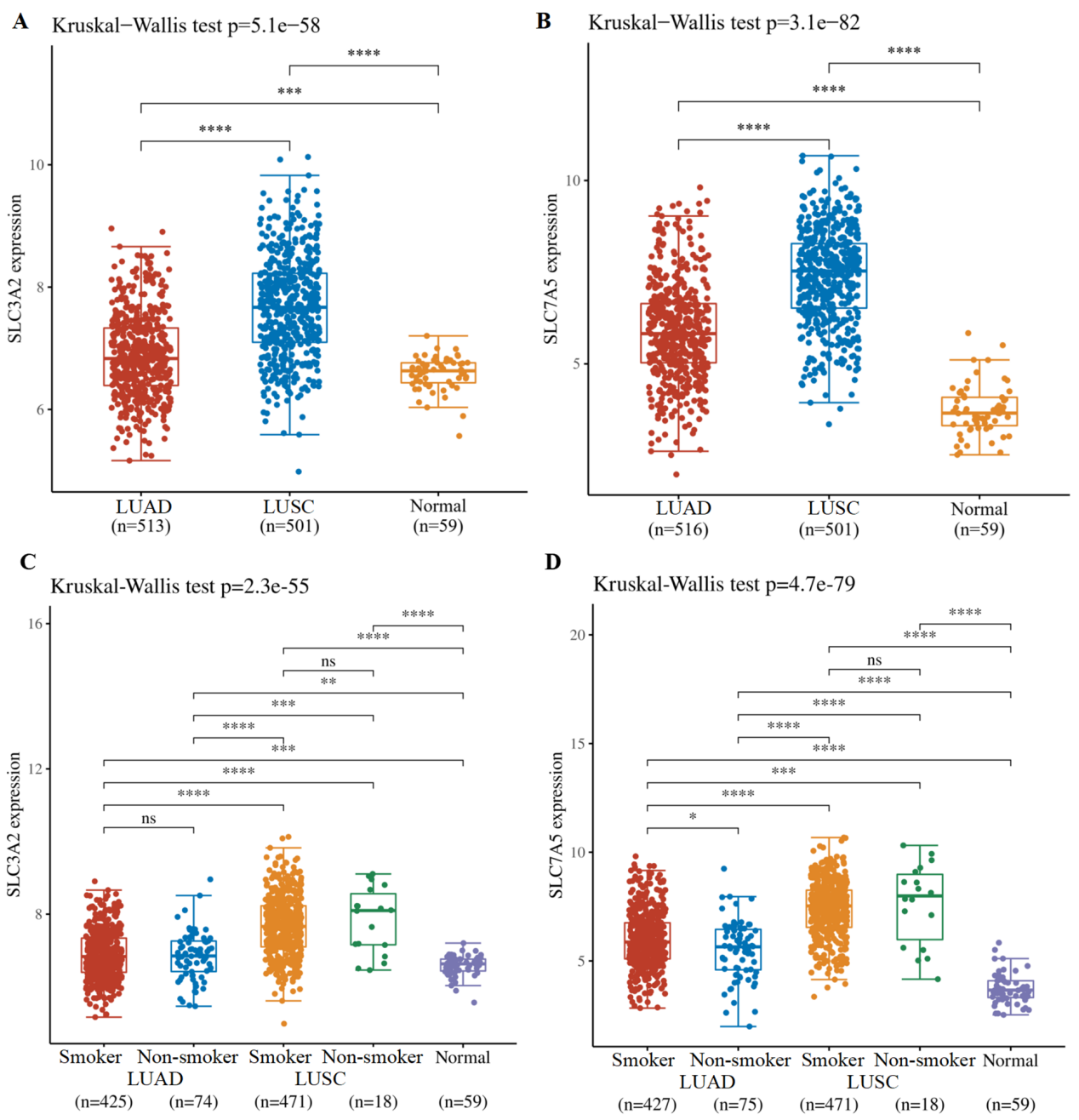
**

**Figure S1. Expression of SLC3A2 and SLC7A5 in LUAD and LUSC patients.** (A) SLC3A2 and (B) SLC7A5 expression were evaluated in primary cancer tissues and matched normal tissues of LUAD and LUSC patients using R software v4.0.3. (C) SLC3A2 and (D) SLC7A5 expression were evaluated in LUAD and LUSC patients with different smocking status using R software v4.0.3.


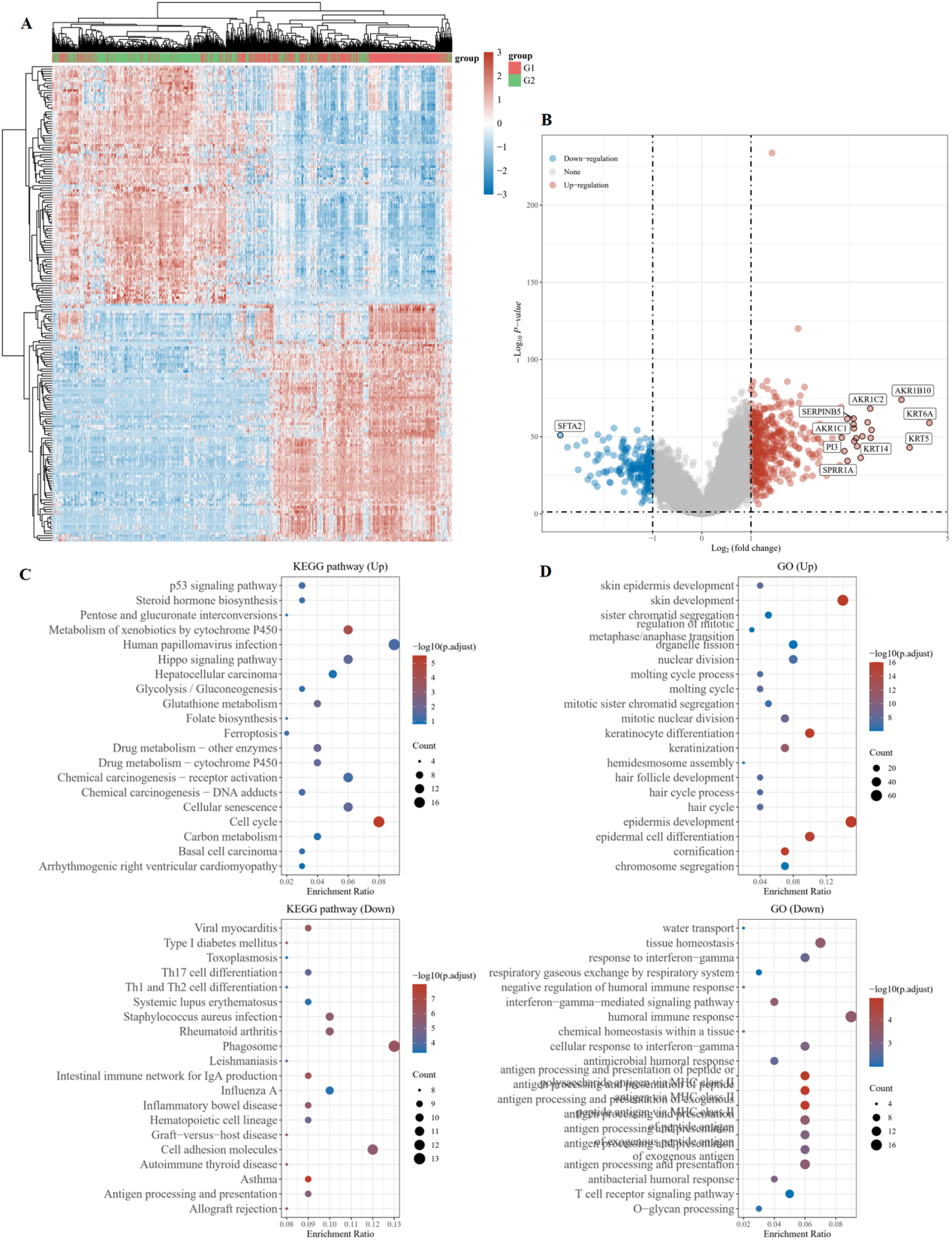


**Figure S2.** **Differentially expressed gene in SLC3A2 high and low expression NSCLC tissues.** (A) Heatmap of differentially expressed genes. (B) Volcano map of differentially expressed genes. (C) KEGG enrichment analysis of differentially expressed genes. (D) GO enrichment analysis of differentially expressed genes.


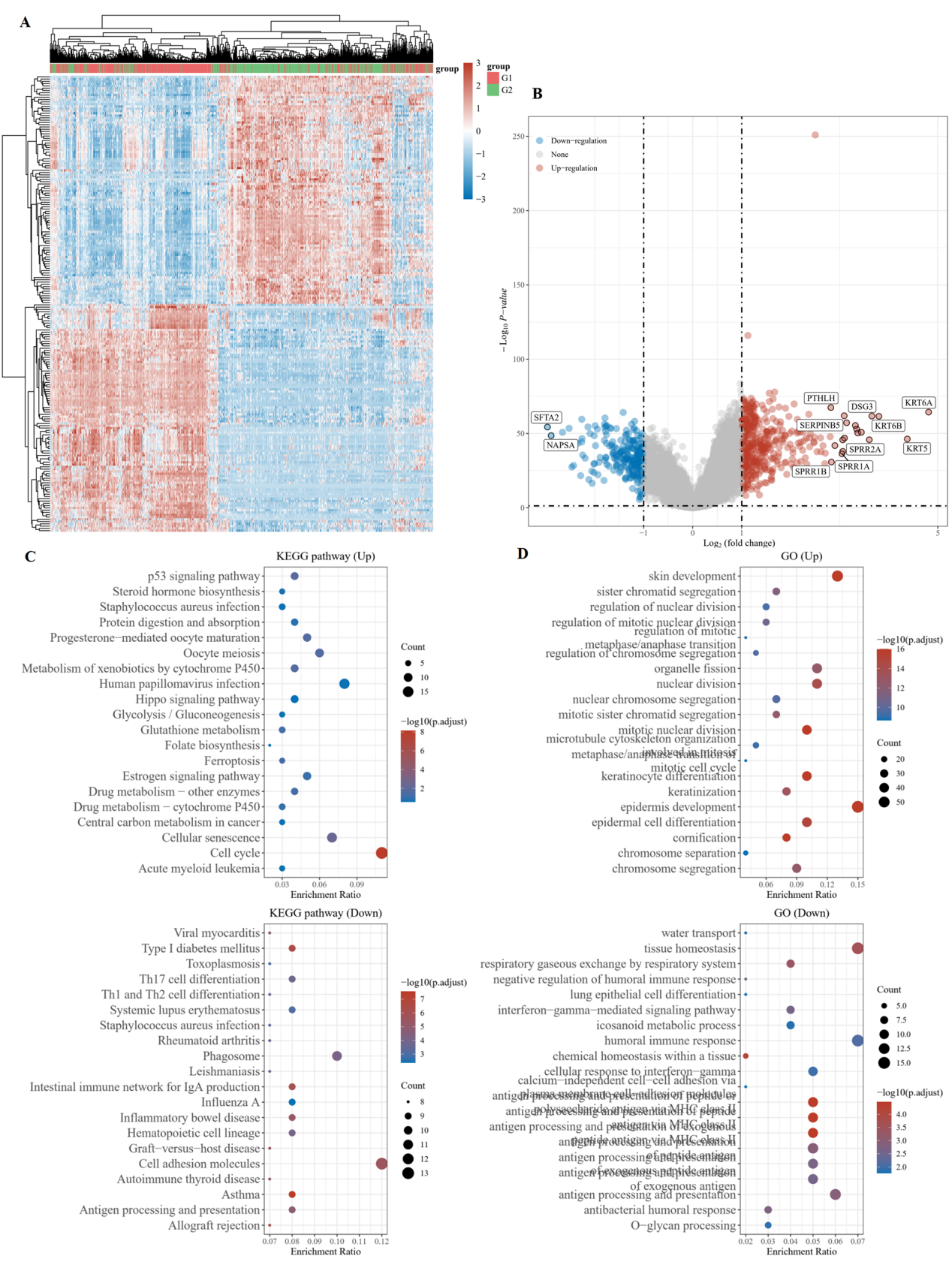


**Figure S3. Differentially expressed gene in SLC7A5 high and low expression NSCLC tissues.** (A) Heatmap of differentially expressed genes. (B) Volcano map of differentially expressed genes. (C) KEGG enrichment analysis of differentially expressed genes. (D) GO enrichment analysis of differentially expressed genes.


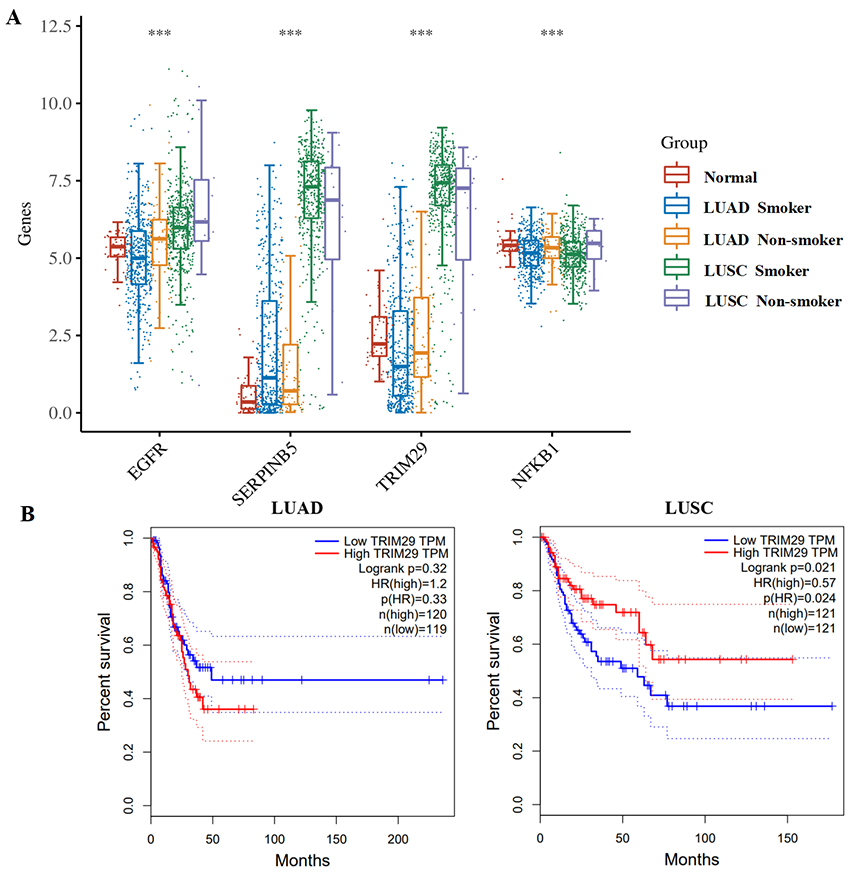


**Figure S4.** **Correlation genes of SLC7A5 in smocking and non-smoking LUAD and LUSC patients.** (A) Expression levels of SLC3A2 and SLC7A5 correlated genes in LUAD and LUSC tissues. (B) Kaplan-Meier analysis was used to analyze the roles of TRIM29 in LUAD and LUSC patients.


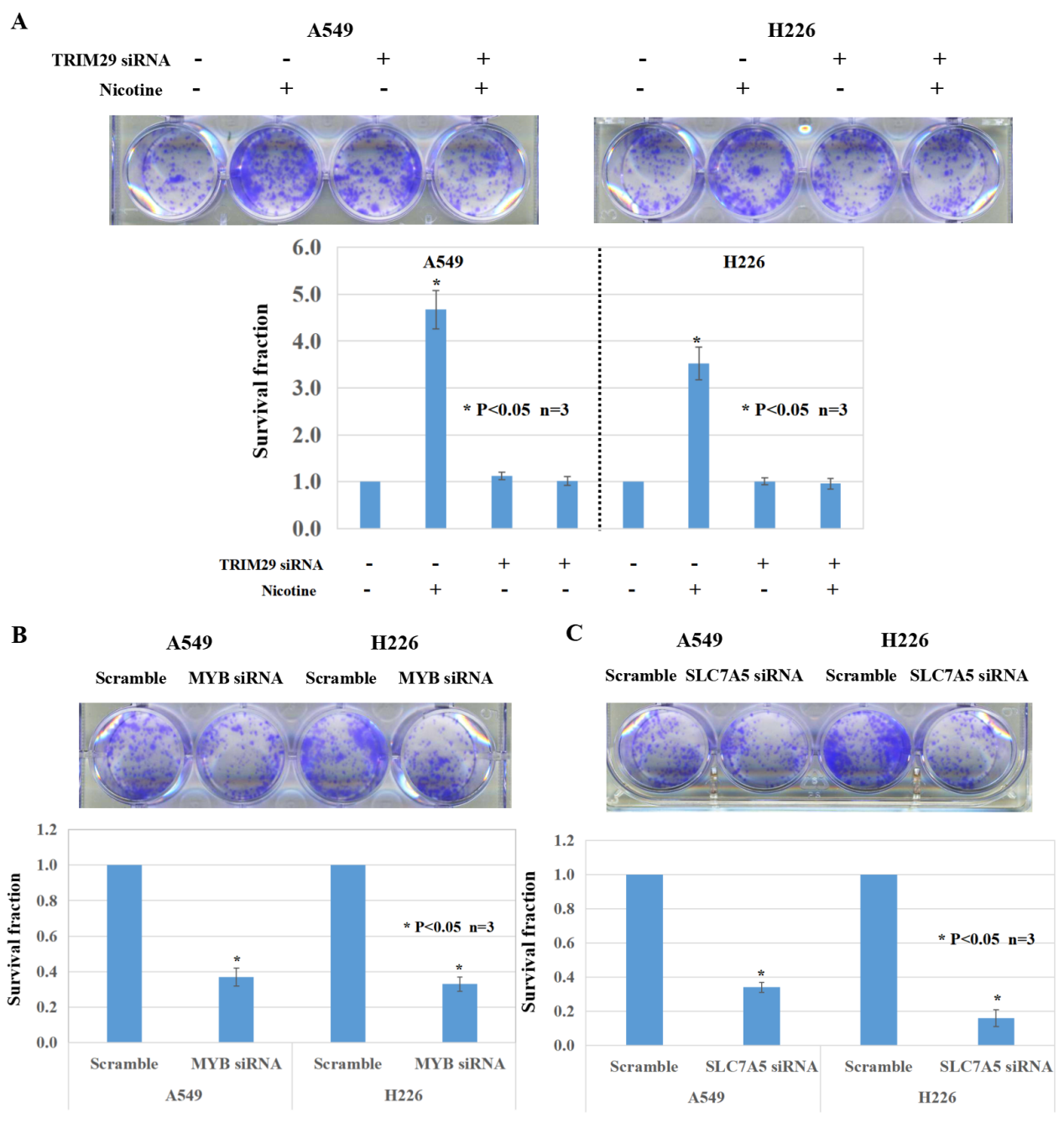


**Figure S5.** **The roles of nicotine-regulated SLC7A5 expression *in vitro*.** (A) Colony numbers of TRIM29 knockdown A549 cells and H226 cells and their parental cells with nicotine treatment. (B) Colony numbers of MYB knockdown A549 cells and H226 cells and their parental cells. (C) Colony numbers of SLC7A5 knockdown A549 cells and H226 cells and their parental cells.


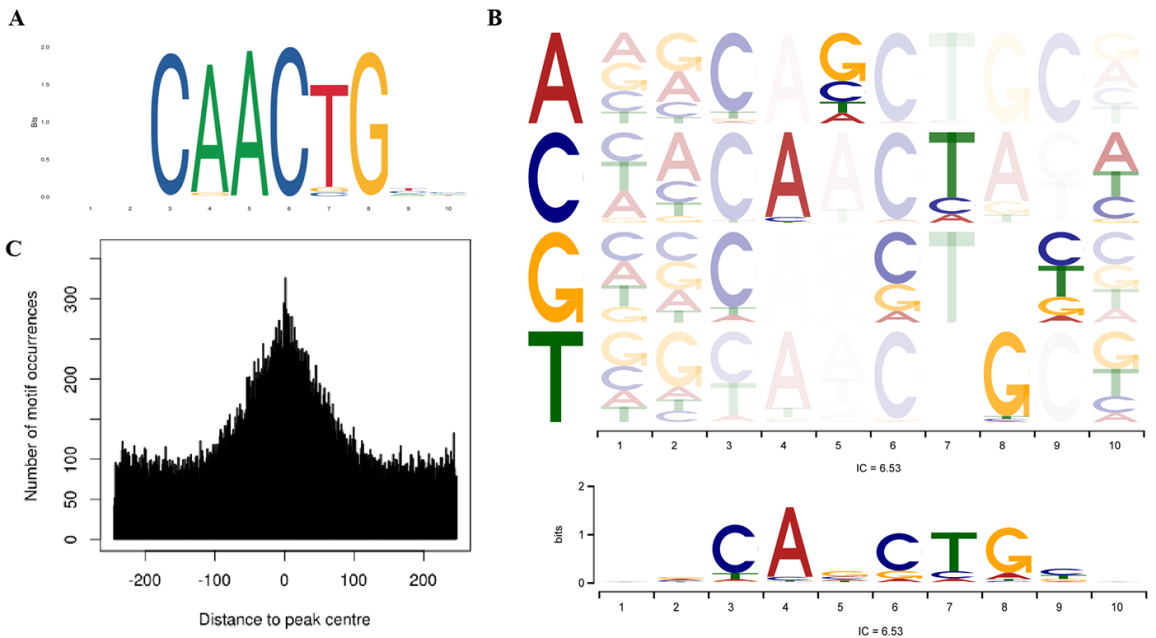


**Figure S6.** **Transcriptional regulation of SLC7A5 by MYB.** (A) DNA motif for SLC7A5 was obtained from JASPAR software. (B) Binding sites of SLC7A5 and MYB were predicted using JASPAR software. (C) SLC7A5 motifs in ChIP-Seq dataset exhibit enrichment around the peakMax, with high scoring motifs distinct from the majority of scores.
